# ALK-positive inflammatory myofibroblastic tumor in the pelvis of a child: a case report and literature review

**DOI:** 10.3389/fonc.2026.1729014

**Published:** 2026-01-21

**Authors:** Lei Yang, Zhiheng Yan

**Affiliations:** 1 First Clinical Medical College, Gansu University of Chinese Medicine, Lanzhou, Gansu, China; 2 Department of Ultrasound, Gansu Provincial Central Hospital, Lanzhou, Gansu, China

**Keywords:** ALK, case report, inflammatory myofibroblastic tumor, pediatric, pelvis

## Abstract

Inflammatory myofibroblastic tumor (IMT) is a rare neoplasm that primarily affects children and young adults. While typically found in the lungs, liver, and gastrointestinal tract, pelvic involvement is recognized but occurs less frequently than intra-abdominal IMT, particularly in pediatric patients. Here we report a case of a 2-year-old boy who presented with a brief history of vomiting and decreased appetite. Imaging revealed a cystic-solid mass in the pelvis with progressive enhancement of the solid component, leading to suspicion of a vascular soft tissue neoplasm. Surgical exploration identified a free-floating mass within the abdominal cavity supported by a long vascular pedicle originating from the splenic hilum, an atypical anatomical finding that added complexity to preoperative diagnosis. Complete surgical resection was performed, and postoperative examination was conducted. Histopathological analysis confirmed IMT, and fluorescence *in situ* hybridization (FISH) detected ALK gene rearrangement, which supported diagnostic confirmation of IMT in this case rather than guiding therapeutic intervention. The patient recovered uneventfully following surgery, with no evidence of recurrence during follow-up. This case supports considering IMT in pediatric pelvic masses and reinforces that complete surgical resection remains the primary treatment. Although ALK gene rearrangement was not associated with therapeutic intervention in the present case, its identification remains diagnostically relevant and may provide important insights into management decisions in selected clinical scenarios, such as recurrence or unresectable disease.

## 1 Introduction

Inflammatory myofibroblastic tumor (IMT) is an intermediate (rarely metastasizing) neoplasm composed of myofibroblastic and fibroblastic spindle cells, accompanied by an inflammatory infiltrate consisting of plasma cells, lymphocytes, and eosinophils (1, 2). A characteristic feature of IMT is a genetic translocation involving the anaplastic lymphoma kinase (ALK)-receptor tyrosine kinase gene located at the 2p23 locus, resulting in aberrant ALK protein overexpression (3). This alteration is reported in approximately 50% of IMT cases, reflecting the significant role of ALK rearrangements in the tumor’s pathogenesis (4, 5). The fusion partner genes vary, with TPM3 and TPM4 among the most common (6). Additionally, receptor tyrosine kinase gene fusions occurring in ALK-negative IMTs include ROS1, NTRK3, PDGFRB, and RET, and a subset of IMTs, notably the more aggressive epithelioid variant, harbor ETV6-NTRK3 fusions (7, 8).

IMTs primarily occur in the lungs, liver, and gastrointestinal tract. Less commonly, these tumors have been reported in the epiglottis, hypopharynx, maxillary sinus, and oral cavity (9). Complete surgical excision with negative margins remains the preferred treatment and reduces the risk of recurrence (10). Although IMTs frequently arise in the abdominal cavity in young children, reported pediatric pelvic IMTs remain limited, and diagnostic uncertainty persists due to overlapping radiologic features with other soft-tissue masses (11, 12). This report describes a rare pediatric pelvic IMT accompanied by an ALK mutation, providing valuable insights for research on this uncommon presentation.

## 2 Case presentation

A 2-year-old boy was admitted to the hospital with a 3-day history of vomiting gastric contents and decreased appetite. He had no history of fever, cough, abdominal pain, diarrhea, jaundice, or urinary symptoms. Growth and developmental history were unremarkable.

On physical examination, the patient was in an acceptable general condition with stable vital signs. Abdominal examination revealed a flat abdomen and a firm, highly mobile, non-tender mass measuring approximately 6 × 5 cm, extending from the umbilicus to the perineum. No abdominal wall varices or peritoneal irritation were observed. The liver and spleen were not palpable, bowel sounds were normal, and no peripheral lymphadenopathy or lower limb edema was detected. Other systemic examinations were unremarkable.

Laboratory investigations revealed the following results: alpha-fetoprotein, 0.72 ng/mL; carcinoembryonic antigen, 1.94 ng/mL; neuron-specific enolase, 19.2 ng/mL (elevated); carbohydrate antigen CA-125, 42.39 ng/mL (elevated); carbohydrate antigen CA19-9, 5.56 ng/mL; serum ferritin, 100 ng/mL. Electrolyte levels and organ function test results were within normal ranges.

**Figure 1:**
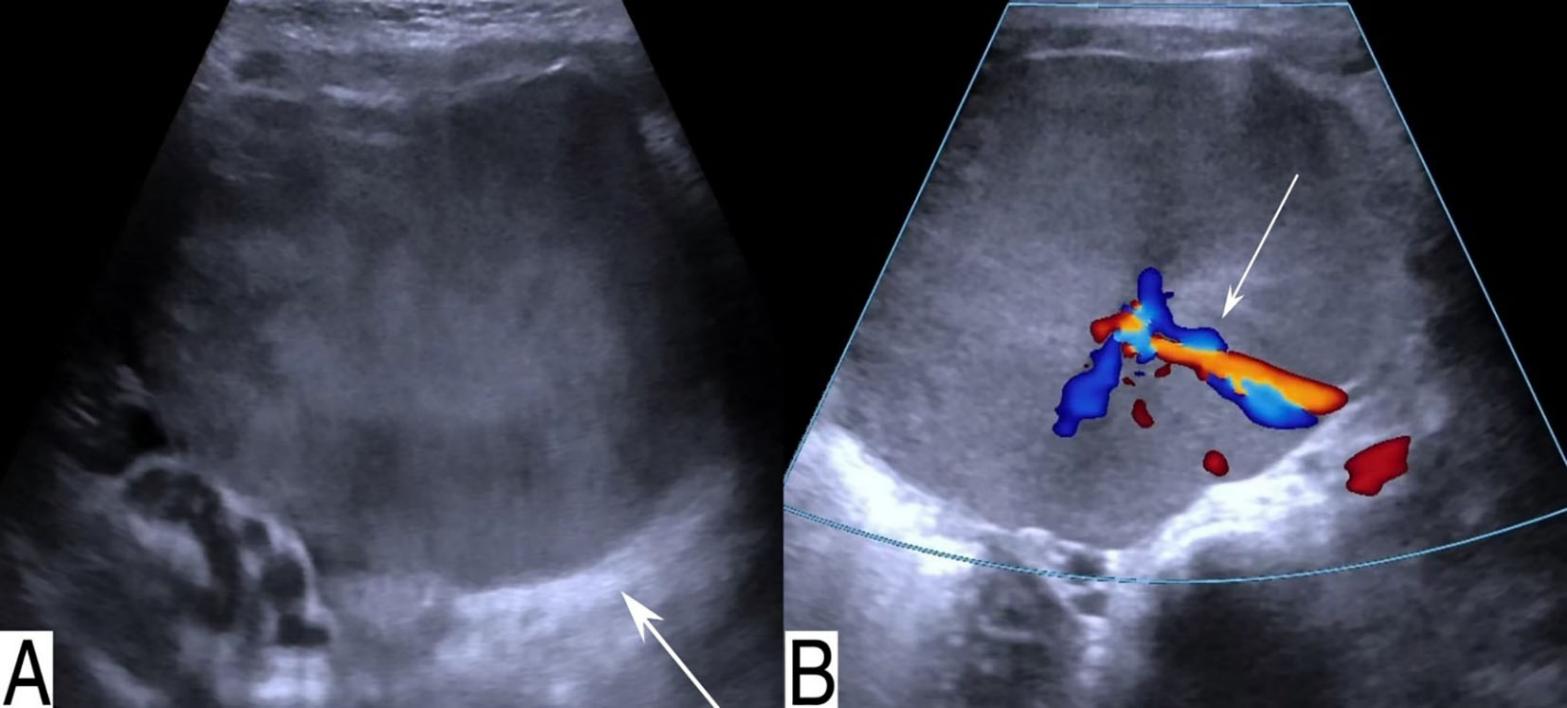
Conventional ultrasound. **(A)** Transverse ultrasound image shows a mass with mixed moderate-to-low echogenicity within the cavity (white arrow), displaying regular contours and clear boundaries. **(B)** Color Doppler imaging reveals short, strip-like blood flow signals within the lesion (white arrow).

**Figure 2:**
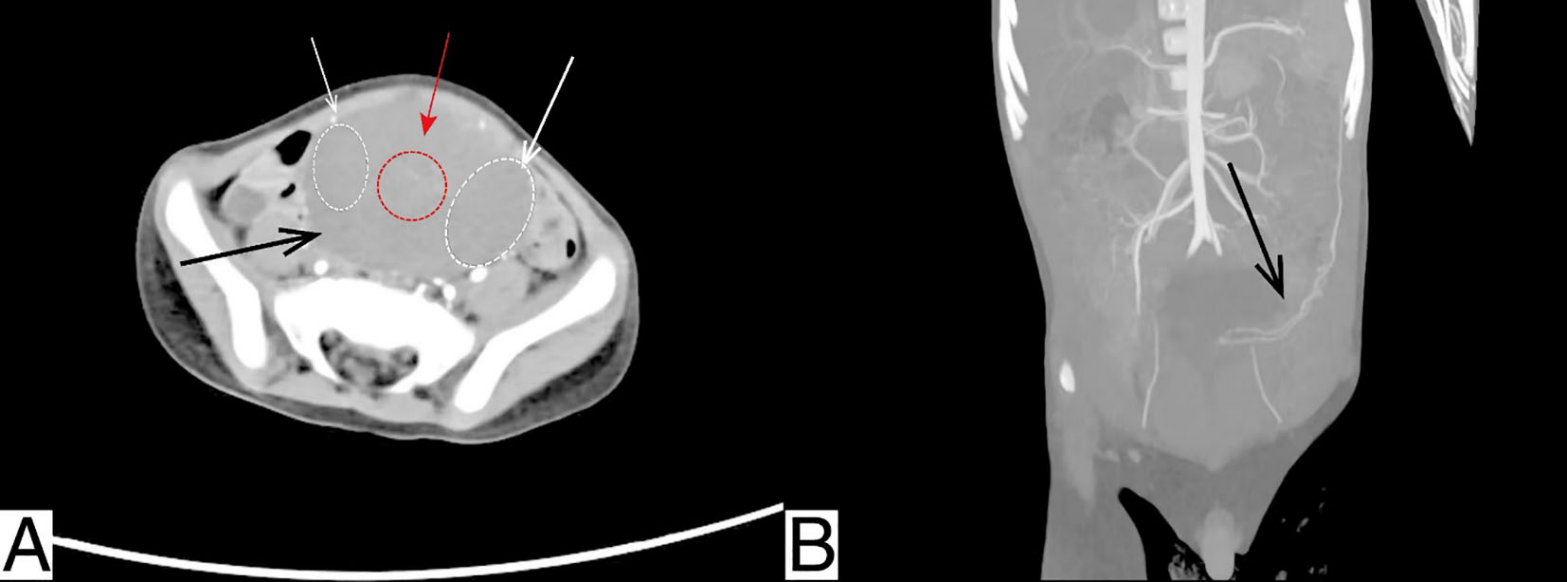
Contrast-enhanced abdominal CT. **(A)** Contrast-enhanced CT image demonstrates a mixed cystic–solid pelvic mass (black arrow). White arrows and dashed outlines indicate the cystic components, while the solid component, showing heterogeneous progressive enhancement, is indicated by the red arrow and dashed outline. **(B)** Arterial phase imaging demonstrates the lesion’s feeding artery (black arrow) originating from a branch of the splenic artery and supplying the mass.

Abdominal ultrasound revealed a well-defined mixed cystic–solid mass measuring approximately 6.8 × 4.4 cm in the pelvis, with regular contours and heterogeneous moderate-to-low internal echogenicity (**Figure 1A**). Color Doppler flow imaging (CDFI) demonstrated short, strip-like internal blood flow signals (**Figure 1B**). Enhanced abdominal CT showed a slightly hypodense mass measuring 6.7 × 4.7 × 7.0 cm, located slightly to the left of the midline in the lower abdomen and pelvis. The solid components exhibited heterogeneous progressive enhancement (**Figure 2A**). A feeding artery arising from a branch of the splenic artery was identified in the arterial phase (**Figure 2B**). The lesion displaced adjacent intestinal loops.

Pelvic MRI further characterized the lesion as a mixed cystic-solid mass measuring approximately 7.6 × 4.7 × 7.5 cm. The solid components demonstrated low-to-intermediate signal intensity on T1-weighted imaging (T1WI), heterogeneous high signal intensity on T2-weighted imaging (T2WI), and high signal intensity on diffusion-weighted imaging (DWI) sequences, with apparent diffusion coefficient (ADC) values of approximately 1.789–2.641 × 10^-3^ mm^2^/s. Progressive enhancement was observed in the solid portions, whereas cystic components showed no enhancement. No enlarged lymph nodes or abnormalities were identified in other solid organs on imaging.

**Figure 3:**
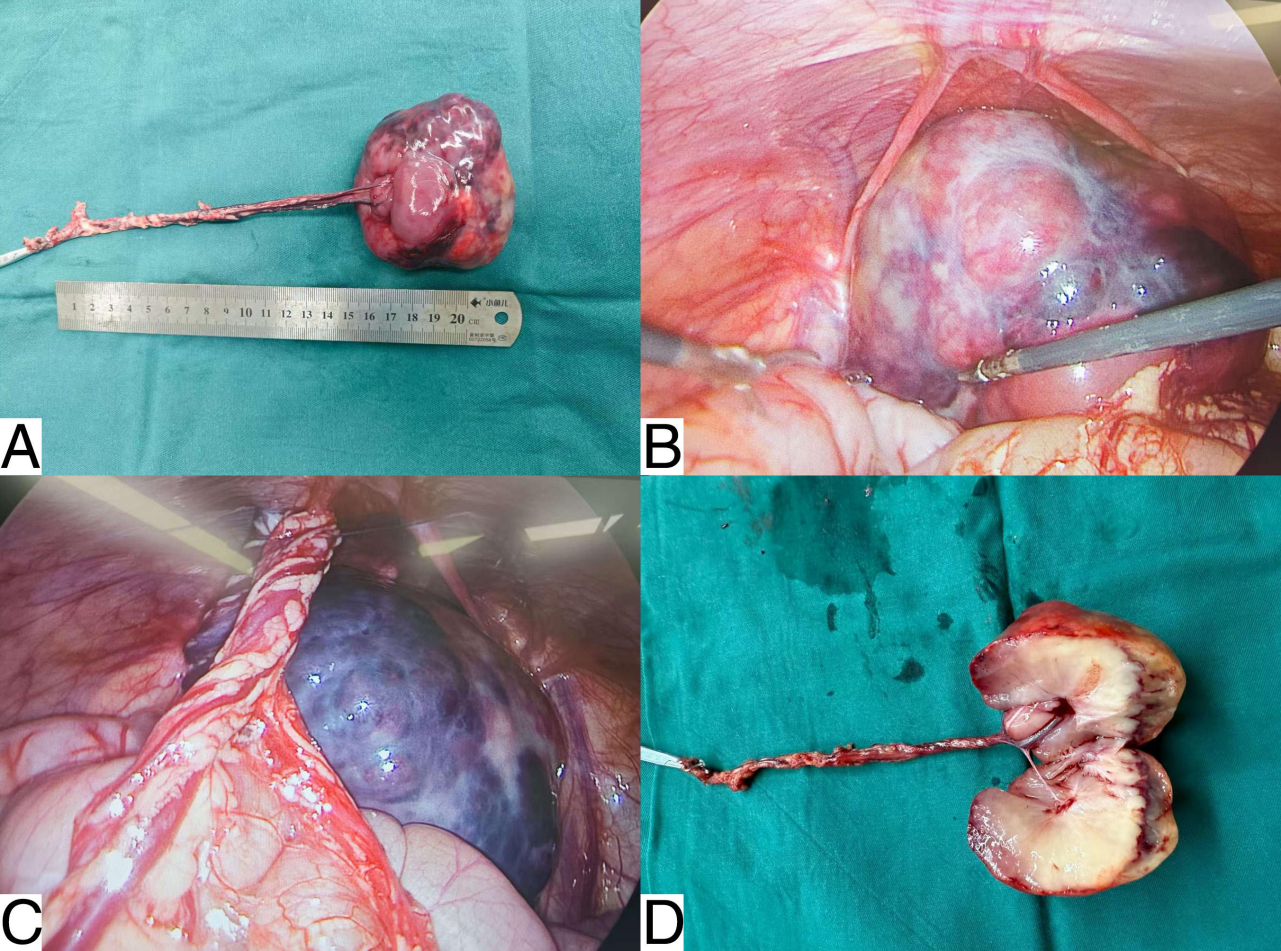
Intraoperative pathological specimen. **(A)** Gross examination of the specimen shows a mass (approximately 6 × 7 cm) with an intact capsule, firm texture, and smooth margins. **(B)** The mass was freely mobile within the abdominal cavity, without adhesions to intestinal loops or the abdominal wall, and is entirely enclosed by a fibrous capsule. **(C)** A vascular pedicle, approximately 10–12 cm long, originated from the splenic hilum, crossed the splenic flexure of the colon, and connected to the tumor. **(D)** Gross pathology revealed a solid, gray-white mass.

The patient underwent abdominal surgery. Intraoperatively, a mass measuring approximately 6 × 7 cm was identified, with a firm texture, intact capsule, and smooth surface (**Figure 3A**). The tumor was free-floating within the abdominal cavity without adhesions to the intestinal loops or the abdominal wall. A vascular pedicle approximately 10–12 cm long originated from the splenic hilum, crossed the splenic flexure of the colon, and connected to the tumor. The vascular pedicle was twisted and adherent to the greater omentum, with collateral circulation maintaining the vascular supply (**Figures 3B–D**). The mass was completely resected and sent for pathological examination.

**Figure 4:**
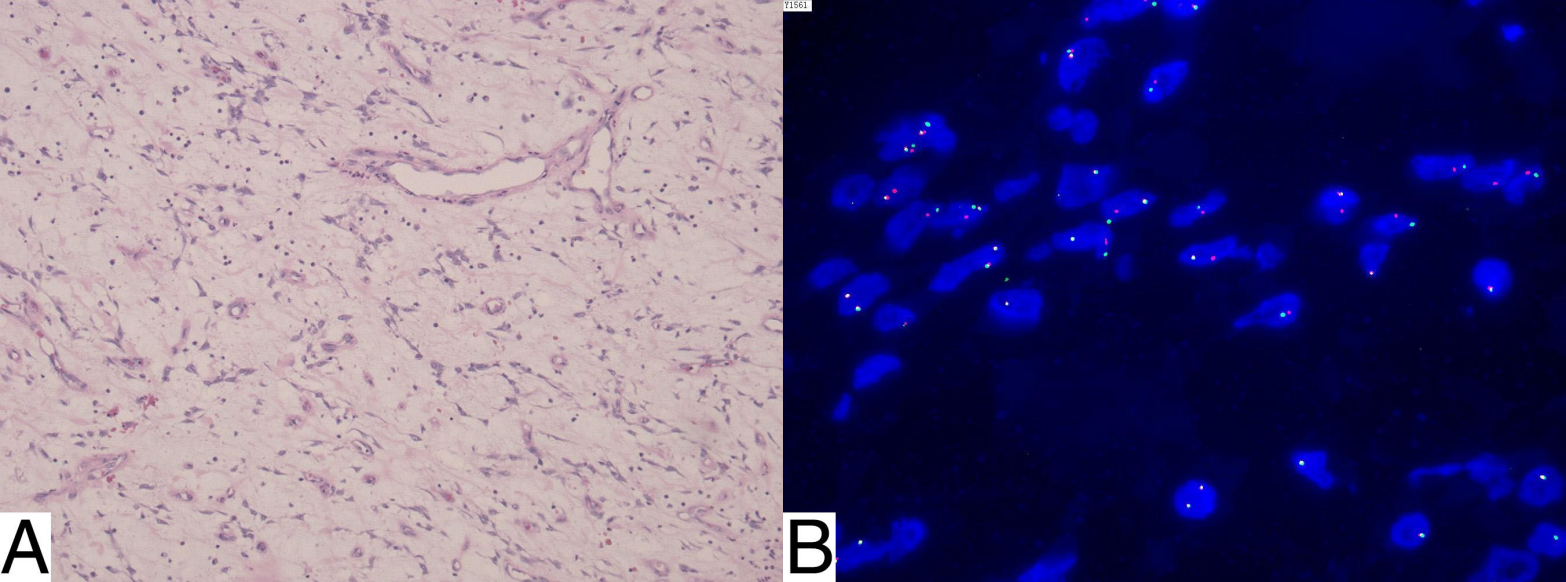
Histology and ALK rearrangement of the pelvic mass. **(A)** Hematoxylin and Eosin (H&E) staining shows spindle-cell proliferation with a myxoid stroma and lymphoplasmacytic infiltration, features consistent with IMT, requiring confirmation with immunohistochemical and molecular testing. **(B)** FISH demonstrates ALK gene rearrangement, with 76% of analyzed nuclei testing positive.

Microscopic examination of the resected specimen revealed spindle-cell proliferation arranged in fascicles within a myxoid stroma and accompanied by lymphoplasmacytic inflammatory infiltrates, features characteristic of IMT, prompting additional immunohistochemical and molecular evaluation for confirmation. Histopathological analysis confirmed that the morphological and immunohistochemical features were consistent with an IMT (**Figure 4A**).

To further characterize the lesion and differentiate it from other spindle-cell tumors, immunohistochemical (IHC) staining was performed. IHC findings were as follows: BCOR (weak +), TRK (-), CD31 (vascular +), CD34 (vascular +), S100 (-), SMA (-), Desmin (+), Ki-67 (proliferative index 10%), CDK4 (weak +), MDM2 (weak +), MUC2 (-), CKP (+), DOG-1 (-), CD117 (-), INI (+), BRG1 (+), MyoD1 (-), Myogenin (-), Myoglobin (-), ALK (+), NF (-), CD45 (scattered +), Vimentin (+), HMB45 (-), MelanA (-), ROS1 (-). Among these IHC profiles, co-expression of desmin and vimentin together with ALK positivity supports myofibroblastic differentiation, a defining feature of IMT (13). Conversely, negative S100 staining strongly argues against neural/neuroectodermal tumors (14, 15). The absence of MyoD1 and myogenin rules out rhabdomyosarcoma (16). In addition, negative DOG-1 and CD117 help exclude gastrointestinal stromal tumor (GIST) (17), and negative HMB45/MelanA excludes PEComa (18). Overall, the IHC profile strongly supports the diagnosis of IMT and effectively rules out major differential diagnoses.

Genetic testing of the pathological tissue using fluorescence *in situ* hybridization (FISH) revealed an ALK gene rearrangement, with 76% of analyzed cells testing positive (**Figure 4B**). To provide a clear summary of the clinical course from symptom onset through diagnosis, treatment, and follow-up, a timeline of key events is presented in **Table 1**.

**Table 1:** Timeline of clinical course from presentation to follow-up.

Timeline	Event description
Day 1	Symptom onset: vomiting; abdominal CT at local hospital performed: pelvic mass identified
Day 3	Hospital admission for further evaluation
Day 4	Pelvic Contrast-Enhanced Magnetic Resonance Imaging (Pelvic CE-MRI) performed - mixed cystic-solid lesion with progressive enhancement of solid components observed
Day 5	Abdominal Color Doppler Ultrasound performed - a cystic-solid mass with internal vascular flow observed
Day 8	Surgical resection performed - complete removal of free-floating solid mass with a vascular pedicle originating from splenic hilum
Day 16	Final pathology and FISH results reported - confirmed IMT with ALK rearrangement
Day 25	Patient discharged after stable postoperative recovery
Follow-up (1 month)	Patient recovered well; no residual lesion; no complications
Latest follow-up (4 months)	Patient remains disease-free; ongoing surveillance planned.

The patient had an uneventful recovery after surgery. Postoperative imaging demonstrated no residual lesion, and subsequent follow-up evaluations demonstrated no clinical or radiologic evidence of recurrence. No adjuvant therapy was required, and no postoperative complications or adverse events were noted. The patient remains disease-free after 4 months of follow-up and continues under regular surveillance due to the recognized risk of recurrence in ALK-positive IMT.

## 3 Discussion

IMT is a benign, nonmetastasizing proliferation of myofibroblasts characterized by potential recurrence and persistent local growth, resembling fibromatoses in certain aspects (19). Histologically, IMTs show variable spindle-cell proliferations within a myxoid to collagenous stroma. These are accompanied by prominent inflammatory infiltrates consisting predominantly of plasma cells and lymphocytes, with occasional eosinophils and neutrophils (1, 20). Coffin et al. described three fundamental histological patterns often combined within the same tumor: a myxoid-vascular pattern, a compact spindle-cell pattern, and a hypocellular fibrous (fibromatosis-like) pattern (19). Clinically, patients with abdominopelvic IMT typically present with nonspecific symptoms such as abdominal pain, loss of appetite, weight loss, and a palpable mass (21). Laboratory abnormalities may include anemia, thrombocytosis, polyclonal hypergammaglobulinemia, and an elevated erythrocyte sedimentation rate (19).

Despite comprehensive multimodal imaging, IMT demonstrates variable and nonspecific radiologic features that resemble those of other abdominal soft-tissue tumors. Therefore, imaging alone is insufficient for definitive diagnosis, and histopathological examination remains essential (22, 23). Based on the cystic-solid morphology and enhancement pattern in this case, the differential diagnosis included mesenteric cyst, teratoma, and other soft -tissue neoplasms previously reported as diagnostic considerations for IMT (22, 24). Preoperative imaging alone could not confirm a definitive diagnosis; therefore, surgical excision served both diagnostic and therapeutic purposes. Histopathology combined with ALK rearrangement identified by FISH confirmed the diagnosis.

ALK gene rearrangements have emerged as a central pathogenetic driver in a substantial proportion of IMTs, particularly in children, and play an important role in diagnostic confirmation. ALK protein expression is detected by anti-ALK immunohistochemistry in approximately 50% of IMT cases and reliably correlates with ALK gene rearrangements (5). In this case, detection of ALK gene rearrangement by FISH supported the diagnosis of IMT and helped distinguish it from other spindle-cell neoplasms with overlapping histopathological features. Given the complete surgical resection and favorable clinical course without adjuvant or targeted therapy, ALK positivity in this patient was primarily diagnostic rather than therapeutic.

To contextualize the molecular significance of this case and align with existing literature, we reviewed previously reported IMT cases that documented ALK status, tumor location, and patient age group. A summary of representative pediatric and adult cases is presented in **Table 2**, illustrating the diverse anatomical sites of IMT, highlighting the predominance of ALK-positive tumors in younger patients, and emphasizing the rarity of pediatric pelvic IMT. While ALK testing is clinically valuable for diagnostic confirmation, its therapeutic implications should be interpreted in accordance with individual clinical context.

**Table 2:** Summary of previously reported IMT cases categorized by patient age group, tumor location, and ALK fusion status.

Case	Author	Year	Age	Tumor location	ALK status	Notes
1	Butrynski et al. (27) (patient 1)	2010	44-year	Omentum/peritoneum	ALK-positive	Partial response to crizotinib which lasted at least 6 months
2	Butrynski et al. (27) (patient 2)	2010	21-year	Gastric/colonic region	ALK-negative	No clinical response to crizotinib; disease progression
3	Nagumo et al. (36)	2018	17-year	Urinary bladder dome	ALK-positive	Neoadjuvant crizotinib reduced tumor size
4	Ogata et al. (30)	2019	37-year	Retroperitoneal	ALK-positive	Complete response to crizotinib after recurrence
5	Brivio et al. (37) (patient 1)	2019	9-year (relapse age 17)	Elbow primary with lung metastasis	ALK-positive	Complete remission; relapse off therapy; regained remission after treatment restart
6	Brivio et al. (37) (patient 2)	2019	14-year	Bladder wall	ALK-positive	70% tumor reduction after ceritinib therapy, even though treatment had to be stopped due to liver and kidney toxicity
7	Mittal A et al. (38)	2021	3-month	Abdomen/pelvis (subdiaphragmatic, paravesical)	ALK-positive	Near-complete response to low-dose ceritinib
8	Chen et al. (39)	2022	12-74 yrs	Bladder	6 of 8 ALK-positive	Retrospective 11-year review
9	Kavirayani et al. (40)	2023	8-month	Pelvic retroperitoneal region	ALK-positive	Surgical resection; no signs of recurrence

Surgical resection remains the primary treatment, with a favorable prognosis following complete excision of localized tumors (25). Systemic therapy is considered in cases of unresectable, advanced (to avoid mutilating surgery), multifocal, or metastatic disease, utilizing various treatments ranging from steroids to chemotherapy (7, 26). Recent studies reported that ALK inhibitors such as crizotinib may induce tumor regression in selected patients with unresectable or recurrent ALK-rearranged IMT. However, final outcomes vary across cases, and additional interventions are sometimes necessary (27–30). These therapeutic considerations did not apply to this case, as complete surgical excision alone resulted in a favorable clinical course without requiring ALK-targeted therapy. Accordingly, discussion of ALK-targeted therapy in this report serves as contextual reference rather than implying therapeutic relevance in this patient.

Therapeutic targeting of the ALK gene represents an important option in selected malignancies driven by ALK fusion events, where constitutive kinase activation underlies tumorigenesis (31, 32). In IMT, responses to crizotinib occurred primarily in cases of unresectable, recurrent, or progressive disease, with heterogeneous treatment duration and outcomes across studies (31, 33). ALK-directed therapy should therefore be regarded as conditional rather than universal. Given the recognized potential for recurrence and malignant transformation in IMT, long-term follow-up remains essential, particularly during the first 12 months after diagnosis (34, 35).

An additional noteworthy aspect of this case is the presence of several unusual intraoperative findings. These include a long vascular pedicle originating from the splenic hilum, marked tumor mobility within the abdominal cavity, and the establishment of collateral circulation despite partial torsion of the pedicle. Although the precise biological or clinical significance of these features remains uncertain, they may reflect a slow-growing process. This gradual growth could have allowed elongation of the vascular pedicle and development of adaptive collateral blood supply. No definitive association between these anatomical characteristics and tumor aggressiveness, prognosis, or ALK status can be established based on a single case. Therefore, these findings should be interpreted as rare but incidental anatomical features.

This study has inherent limitations related to its single-patient case-based reporting. Specifically, it included only one pediatric patient diagnosed with pelvic IMT accompanied by an ALK mutation, an already rare clinical entity within this demographic. The absence of comparative treatment data and limited long-term follow-up restricts the generalizability of these findings. Nevertheless, this case provides a valuable descriptive example of pediatric pelvic IMTs with ALK mutations. It contributes diagnostic and descriptive insights into an uncommon presentation and may serve as a reference for future clinical observations and research on similar rare cases.

## 4 Conclusion

IMTs can present in the gastrointestinal tract and pelvic region of children and may exhibit atypical clinical and anatomical features. Complete surgical resection remains the treatment of choice for localized disease and was sufficient to achieve a favorable outcome in the present case. Identification of ALK mutations through genetic analysis contributed primarily to diagnostic confirmation rather than therapeutic decision-making in this patient, as no adjuvant or targeted therapy was needed following complete tumor excision. Although ALK-targeted therapies such as crizotinib have demonstrated efficacy in selected patients with unresectable or recurrent ALK-rearranged IMT, their application was not indicated in this case. Assessment of ALK status remains relevant, as it may inform management strategies during advanced disease. Long-term follow-up is essential given the recognized risk of recurrence in IMT.

## Data Availability

The raw data supporting the conclusions of this article will be made available by the authors, without undue reservation.
